# A Longitudinal Study of the Relationship between Shift Work and Prostate-Specific Antigen in Healthy Male Workers

**DOI:** 10.3390/ijerph18147458

**Published:** 2021-07-13

**Authors:** Yesung Lee, Woncheol Lee, Hyoung-Ryoul Kim

**Affiliations:** 1 Department of Occupational and Environmental Medicine, Kangbuk Samsung Hospital, School of Medicine, Sungkyunkwan University, Seoul 03181, Korea; wtb7531@naver.com (Y.L.); doctor.oem@gmail.com (W.L.); 2 Department of Occupational and Environmental Medicine, Seoul St. Mary’s Hospital, College of Medicine, The Catholic University of Korea, Seoul 06591, Korea

**Keywords:** shift work, rotating shift work, prostate cancer, prostate-specific antigen, cohort study, longitudinal study, Kangbuk Samsung Health Study

## Abstract

As shift work has become prevalent globally, it is important to evaluate the health effects of shift work on employees. Several studies have demonstrated a positive association between shift work and prostate cancer. Therefore, we aimed to further examine the relationship between shift work and elevated prostate-specific antigen (PSA). Our study collected data from 66,817 male participants at baseline and followed up for about 6 years. We categorized shift worker status and shift schedule types. To evaluate the risk of elevated PSA on shift workers, we estimated hazard ratios using the Cox proportional hazards regression analyses. During a median follow-up of 4.1 years, 1030 participants developed elevated PSA. The multivariable-adjusted hazard ratio (HR) of elevated PSA for shift workers compared with daytime workers was 1.37 (1.04–1.80). Among shift workers, rotating shift workers (HR = 1.47, 95% CI 1.06–2.03) showed a significantly increased risk of elevated PSA compared with daytime workers. Our longitudinal study provides evidence for an association between shift work, especially rotating shift work, and elevations of PSA.

## 1. Introduction

Globally, about 15–20% of workers are engaged in shift work, which has become increasingly prevalent throughout the world over the last several decades [1,2]. In the USA, 29% of workers in 2015 worked an alternative shift, which is not a regular day shift, and 15% of workers worked a fixed night shift [3]. Similarly, according to Eurofound’s European Working Conditions Survey, approximately 21% of European workers are shift workers [4]. In Korea, the Statistics Research Institute of Statistics Korea reported that around 7.1% of workers had a shift work system in 2015 [5]. As shift workers are common in most industrialized countries, it is important to evaluate the health effects of shift work on employees. Studies have demonstrated that shift work affects cardiovascular disease [6], mood disorders [7], gastrointestinal disease [8], and cancer [9].

In 2007, the International Agency for Research on Cancer (IARC) of the World Health Organization classified shift work involving circadian disruption as “probably carcinogenic to humans” (group 2A) based on sufficient evidence in experimental animals but limited evidence in humans [10]. On the basis of limited evidence, shift work was related to breast cancer, prostate cancer, and colorectal cancer [9]. Especially, breast cancer was strongly associated with shift work among premenopausal women [11]. On the other hand, several studies have shown associations between shift work and prostate cancer, but these studies were few and lacked consistency [9]. To date, despite a limitation of inconsistency, several studies have demonstrated a positive association between shift work and prostate cancer, especially in rotating shift workers [12,13,14], and such a relationship cannot be ignored. In this sense, it is reasonable to evaluate a more temporal relationship between shift work and prostate cancer by a longitudinal study.

Several studies have shown that prostate-specific antigen (PSA) can be used as a biomarker of prostate cancer and provides a potential screening test for the early detection of prostate cancer [15,16]. Although PSA can be affected in many other conditions, such as benign prostatic hyperplasia (BPH), prostatitis, changes in vitamin D, or cardiovascular risk factors, a cut-off value of PSA ≥ 3.0 ng/mL was recommended as an indication for biopsy, and screening of PSA decreased the mortality of prostate cancer in the European Randomised Study of Screening for Prostate Cancer [17,18,19]. Furthermore, studies have demonstrated that an elevation of PSA may precede clinical symptoms of prostate cancer [16,20]. Thus, given the association between shift work and prostate cancer, there may be also a relationship between shift work and PSA.

A study of the National Health and Nutrition Examination Survey reported an association with shift work and elevated PSA levels, which implicates that shift workers had a higher risk of developing prostate cancer [21]. On the other hand, another study of Korean workers in tire manufacturing indicated that night shift work did not show increased PSA levels [22]. Although there have been many studies on the relationship between shift work and prostate cancer, there is still the need for further research because of the inconsistency of the relationship [12,23,24,25]. In this regard, we conducted a study in a different way from the previous studies by identifying the risk of elevated PSA, which is a tumor marker for prostate cancer, in relatively young shift workers with a relatively low incidence of prostate cancer, which has not been studied in a longitudinal study design. Therefore, the present study aimed to examine the relationship between shift work and elevated PSA and to further identify the underlying mechanism while adjusting for various confounding and potential mediating variables that are likely to affect the relationship in a large-scale prospective cohort of young and middle-aged healthy Korean male workers who participated in a health screening program.

## 2. Materials and Methods

### 2.1. Study Population

The Kangbuk Samsung Health Study is a cohort study of South Koreans who were aged 18 years and older and who underwent an annual or biennial comprehensive health screening examination at the Kangbuk Samsung Hospital Total Healthcare Center in Seoul and Suwon, South Korea [26]. Most participants were employees of companies in various industries and local governmental organizations and their spouses. In South Korea, the Industrial Safety and Health Law requires annual or biennial health medical examinations of all employees free of charge. The remaining examinees underwent health medical check-ups voluntarily at the healthcare center.

**Figure 1:**
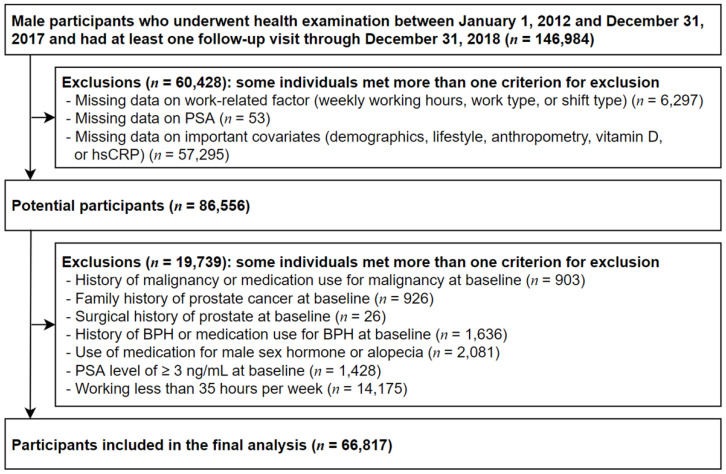
Flowchart of study participants (PSA, prostate-specific antigen; hsCRP, high-sensitivity C-reactive protein; BPH, benign prostatic hyperplasia).

Our study included a total of 146,984 male participants who experienced comprehensive health examinations from 1 January 2012 to 31 December 2017 and had undergone at least one other medical screening examination before 31 December 2018. We excluded 60,428 participants with missing variables at baseline or any follow-up visit. Figure 1 summarizes the work-related factors, PSA, and any other relevant covariates for adjustment. Within the potential participants (*n* = 86,556), we further excluded 19,739 participants who had any of the following conditions at baseline to examine the effects of shift work while excluding those who were susceptible to the increase in PSA or the incidence of prostate cancer: history of malignancy or medication use for malignancy; family history of prostate cancer; surgical history involving prostate; history of BPH or medication use for BPH; medication use for male sex hormone or alopecia; PSA ≥ 3.0 ng/mL; and working less than 35 h per week. Finally, 66,817 participants were eligible for our study at baseline. This study was approved by the Institutional Review Board (IRB) of Kangbuk Samsung Hospital, which waived the requirement for informed consent because we accessed only de-identified data routinely collected as part of health screening examinations (IRB No: KBSMC2021-03-012). The data are not available to be shared publicly, because we do not have permission from the IRB to distribute the data. However, the data can be available from the Kangbuk Samsung Health Study upon reasonable request, whose authors may be contacted through the corresponding author of this manuscript.

### 2.2. Measurements

All examinations were conducted at the Kangbuk Samsung Hospital Total Healthcare Screening Center in Seoul and Suwon. At each visit, the demographic characteristics, smoking status, alcohol consumption, regular exercise, sleep duration, education level, total monthly household income, marital status, medical history, and medication use were collected by using standardized, self-administered questionnaires. Smoking status was categorized as non-, former, and current smokers. Alcohol consumption was categorized as ≥10 g/day and <10 g/day. We assessed the weekly frequencies of moderate- and vigorous-intensity physical activity, which were categorized as <3 or ≥3 times/week, respectively. Education level was categorized as less than college, college graduate, and higher. Total monthly household income was categorized <$6000/month or ≥$6000/month. Sleep duration was assessed by using a following question: “How many hours a day did you actually get sleep during the past month?” Marital status was categorized as unmarried, married, and other (such as divorced, separated, and widowed).

Shift work was identified using the following self-reporting questionnaire: “In the past year, during which time of the day did you work the most?” Daytime worker was defined as the participants who answered that “I worked mostly during the day (between 6 AM and 6 PM).” Shift worker was defined as the participants who answered that “I worked during other hours.” In shift workers, shift schedule types were categorized as fixed evening shift (between 2 PM and 0 AM), fixed night shift (between 9 PM and 8 AM the next day), regular day and night rotating shift, 24 h rotating shift, irregular rotating shift, split shift (working ≥ 2 shifts/day), and the other shifts. Shift workers were subdivided into three subgroups by shift schedule type: fixed shift work (fixed evening and fixed night), rotating shift work (night rotating shift, 24 h rotating shift, and irregular rotating shift), and other shift work (split shift and the other shifts). Weekly working hours were obtained using the following question: “How many hours did you work in a week on average in your job for the past year, including overtime?”

Blood pressure (BP), weight, and height were obtained by trained nurses. Hypertension was defined as a systolic BP ≥ 140 mmHg, a diastolic BP ≥ 90 mmHg, a self-reporting history of hypertension, or medication use for hypertension. Obesity was defined as a body mass index (BMI) ≥ 25 kg/m^2^. Fasting blood measurements consisted of high-sensitivity C-reactive protein (hsCRP), total vitamin D, and PSA. Serum PSA was measured using an electrochemiluminescence immunoassay with the Modular E170 system (Roche Diagnostics, Tokyo, Japan) [18].

### 2.3. Statistical Analysis

The chi-square test and one-way ANOVA were used to compare the characteristics of participants categorized by shift work status (daytime worker vs. shift worker). The primary endpoint was the cut-off value of PSA ≥ 3.0 ng/mL. Study participants were followed up from the baseline to the primary endpoint visit or to the last available visit before 31 December 2018, whichever came first.

Hazard ratios (HRs) and 95% confidence intervals (CIs) for elevated PSA were estimated by using Cox proportional hazards regression analyses. Firstly, we adjusted for age, and Model 1 was adjusted for age, center (Seoul and Suwon), smoking status, alcohol intake, regular exercise, education level, total household income, marital status, and weekly working hours. Model 2 was further adjusted for BMI and hsCRP. To evaluate the mechanism underlying the observed associations, Model 3 was further adjusted for sleep duration and total vitamin D. A proportional-hazards assumption was assessed by examining graphs of estimated log (-log) survival and by using the *‘estat phtest’* command based on Schoenfeld residuals; no violation of the assumption was found. To explore the effects of changes in covariates during the follow-up period, additional analyses were conducted using covariates as time-varying covariates in the models. In addition, to elucidate the associations according to shift schedule type, we estimated HRs and 95% CIs for elevated PSA by dividing shift workers into fixed shift workers, rotating shift workers, and other shift workers. Lastly, stratified analysis in clinically relevant subgroup was performed by age (<40 vs. ≥40 years). Interaction between shift schedule types and subgroup by age was tested using the likelihood ratio test, which compared models with and without multiplicative interaction terms.

Statistical analyses were performed using STATA version 16.1 (StataCorp LP, College Station, TX, USA). All reported *p*-values were two-tailed. A *p*-value < 0.05 was considered statistically significant.

## 3. Results

In Table 1, the mean (standard deviation) age and weekly working hours at baseline were 37.2 (6.4) years and 51.8 (9.2) hours, respectively. Shift work was associated with age, sleep duration, alcohol intake, regular exercise, high education level, high household income, marital status, BMI, and total vitamin D.

In Table 2, the relationship between shift work and elevated PSA is given. Among 66,817 subjects, a total of 269,447.2 person-years were obtained during the follow-up period (median follow-up, 4.1 years; interquartile range, 2.5–5.6 years). There were 1030 incident cases of elevated PSA within the follow-up period. All models demonstrated that shift work was associated with a significantly increased risk of elevated PSA compared with daytime work. The multivariable-adjusted HR (95% CI) of elevated PSA for shift workers was 1.37 (1.04–1.80) in Model 3. When we introduced confounders as time-varying covariates, the association between shift work and elevated PSA still significantly remained in the time-dependent model.

Table 3 presents the association between various shift schedule types and elevated PSA. Especially for rotating shift work, all models showed that shift work was significantly associated with elevated PSA, even after introducing the time-dependent model. For fixed (HR = 1.09, 95% CI 0.41–2.90) and other shift workers (HR = 1.22, 95% CI 0.72–2.07), the HRs were increased but the associations were shown to be insignificant.

In subgroup analysis (Table 4), the association between rotating shift workers and elevated PSA was significant among participants with age ≥40 years (HR = 1.74, 95% CI 1.02–2.97). There was no interaction with the predetermined subgroup.

## 4. Discussion

In our longitudinal study, the results demonstrated that shift workers had a significantly higher risk of increased PSA. Even after adjusting for relevant covariates including age, smoking, and exercise and introducing a time-dependent model, the association was significant with consistently unchanged HRs. When we divided shift workers by schedule type, such as fixed, rotating, and other shift workers, the association with the potential risk of prostate cancer was only significant for rotating shift workers.

There have been several systematic reviews regarding the relationship between shift work and prostate cancer [14,24,25,27]. However, there were several limitations in previous studies. One limitation that most systematic reviews have pointed out was the lack of Asian longitudinal studies, resulting in the small study effect [14,24,25]. Only two Japanese longitudinal studies have been included in most studies [28,29]. On the other hand, our study is the first prospective cohort Korean study with the largest sample size in Asia (N = 66,817). Moreover, other limitations were that there is a lack of studies dealing with the association between rotating or fixed shift work separately and prostate cancer [14], and that it is insufficient to consider various confounding factors [25,27]. However, the present study not only addressed the effect of rotating shift or fixed shift work but also included and analyzed more variables than any other previous studies. Lastly, one of the suggested pieces of evidence for the association between shift work and prostate cancer was the cross-sectional study of elevated PSA with shift workers [21,27]. From this point of view, our first longitudinal study analyzing the relationship between shift work and elevated PSA is necessary to establish evidence for the risk of prostate cancer in shift workers.

Although PSA is considered a tumor marker of prostate cancer, there are several factors affecting PSA level. Non-cancerous diseases such as BPH, prostatitis, and urinary tract infection can increase PSA [30]. However, most participants of our study were asymptomatic with young and middle-aged healthy workers without known prostatic diseases at baseline who had undergone health examinations including serum PSA measurements as a routine screening test, which made our cohort less affected by selection bias related to the comorbidities of the older population. Moreover, recent studies have reported that PSA was inversely associated with obesity and cardiovascular conditions, which are also adverse effects of shift work [6,18,31]. Hence, we adjusted potential confounders including BMI and hsCRP, which is known as an inflammatory and cardiovascular marker. However, the association between shift workers and elevated PSA still remained significant.

In an Italian cross-sectional study, shift workers had a vitamin D deficiency compared with daytime workers [32]. On the other hand, studies have demonstrated that a high vitamin D level was associated with inhibited prostate carcinogenesis, decreased mortality of prostate cancer, and decreased PSA levels [19,33,34]. However, there is a controversy about the effect of vitamin D supplements on preventing prostate cancer [35,36]. Meanwhile, shift work is associated with sleep restriction [37], and several studies have reported that sleep duration was inversely associated with prostate cancer by disrupting the circadian cycle, which results in the dysregulation of clock genes related to tumor suppression [38,39], whereas there is also a controversy about a lack of clear evidence [40]. In this regard, we considered vitamin D and sleep duration as a mediator of the association between shift work and prostate cancer. However, when adjusted for sleep duration and total vitamin D, the significance was still observed, suggesting that there could be other mechanisms explaining our study findings.

The present study showed that rotating shift workers, but not fixed shift workers, were associated with a higher risk of increased PSA compared with daytime workers. This result was in line with previous studies indicating that rotating shift workers were more prone to have prostate cancer [13,14]. Mancio et al. explained that more severe disruption of the circadian rhythm due to insufficient time for the circadian clock to adapt and consequently lower melatonin levels during the sleep period for rotating shift workers than fixed shift workers result in a higher risk of prostate cancer [14]. Razavi et al. also explained that a worse alignment of shift schedule and chronotype may disrupt the melatonin rhythms of the sleep–wake period [41]. In addition, Xi et al. reported that melatonin may suppress the prostate cancer cell (LNCaP cell) through the melatonin receptor (MT1 receptor) with attenuated calcium influx, which activates protein kinase C to inhibit the PSA gene (KLK3 gene), consequently decreasing PSA levels [42,43]. From this point of view, it seems reasonable that a lack of melatonin results in increased PSA levels. Additionally, PSA could be involved in the pathogenesis of prostate cancer by interacting with the androgen receptor target gene and stimulating oxidative stress in prostate cancer cell [44]. Since the mechanisms underlying the association between shift workers and PSA are not fully understood, further studies to elucidate the association are needed. However, in our cohort, since the number of fixed shift workers and incident cases of fixed shift workers were relatively small, the association needs to be carefully interpreted, and further studies may show different results.

Considering that our study population was comprised of relatively young and middle-aged workers, and that a recent study by Bleryer et al. reported that the incidence rate of prostate cancer is increasing in young adults classified as adults with age <40 years [45], we conducted stratified analysis based on age of 40. The risk of elevated PSA was significantly higher in rotating shift workers ≥40 years but not in those <40 years, with no interaction. Since the incidence of prostate cancer increases rapidly from the age of 40s to 50s [45] and shows a relatively low prevalence in Asians compared to Westerners [46], the result of the risk of elevated PSA being insignificant among participants <40 years was consistent with previous studies. Based on our study, it is reasonable to perform a PSA test on shift workers from the age of 40 or older. However, as our findings may not be generalized to other populations, the cut-off age needs to be determined through further well-designed studies.

There are several limitations of the present study that need to be considered. First, our primary outcome was elevated PSA but not prostate cancer cases. PSA can be increased in various conditions such as BPH, prostatitis, trauma, and infection. As our study population consisted of mostly young and middle-aged, healthy workers with no prostate disease at baseline, participants with increased PSA could be considered at high risk for prostate cancer. Although, to date, the association between shift workers and prostate cancer is still controversial, our study results show that shift workers, especially rotating shift workers, had a higher risk of elevated PSA compared with daytime workers, providing more strong evidence for the association. Hence, this study could very well be the basis for preventing prostate cancer by performing a PSA test in health screening examinations for shift workers. Second, when we collected the study population, a large number of participants were excluded due to missing data, which can produce selection bias. However, when we compared our eligible participants with excluded participants, the baseline characteristics such as age, BMI, total vitamin D, and PSA were almost the same (data not shown). Thus, the bias could be minimized and would not have a significant effect on our study results. Third, free PSA and histology were not evaluated in our health screening examination. However, a free PSA test is not routinely performed for screening examinations in Korea. Since a biopsy is an invasive test, it cannot be done for screening, as it can cause unnecessary harm and unintended complications to healthy individuals. Otherwise, a PSA test is widely used as a tumor marker and the results can be easily obtained in a large-scale population for analysis. Fourth, when performing statistical analysis on shift work, other time-related factors, such as chronotype and daily shift working hours, were not considered. Instead, weekly working hours were adjusted for the analysis, and the risk was not changed significantly.

Notwithstanding the limitations, the current study had some notable strengths. To our knowledge, this cohort study is the first longitudinal study to show the relationship between shift work and the risk of elevated PSA. Furthermore, our study results were reliable due to the large-scale sample size and refined cohort design with standardized result data. Finally, our study was based on a relatively young and middle-aged healthy population, resulting in less susceptibility to survivor bias from comorbidities.

## 5. Conclusions

In our large-scale cohort study, shift workers, especially rotating shift workers, were associated with a higher risk of increased PSA, which can be considered as a potential screening tool for prostate cancer in shift workers. Further studies are needed to elucidate the exact mechanisms underlying the association.

## Figures and Tables

**Table 1:** Baseline characteristics of study participants by shift work status.

		Shift Work Status		*p*-Value ^§^
Characteristics	Overall	Daytime Worker	Shift Worker	
Number	66,817	62,147	4670	
Age (years) *	37.2 (6.4)	37.5 (6.4)	33.0 (5.6)	<0.001
Working hours (hours/week) *	51.8 (9.2)	51.8 (9.1)	51.5 (10.3)	0.068
Sleep duration (hours/day) *	6.2 (1.0)	6.2 (1.0)	6.3 (1.1)	<0.001
Ever smoker (%)	72.5	72.8	68.7	<0.001
Alcohol intake (%) ^a^	56.1	55.8	60.0	<0.001
Regular exercise (%) ^b^	13.3	13.0	16.8	<0.001
High education level (%) ^c^	90.4	92.0	69.4	<0.001
High household income (%) ^d^	33.3	34.7	15.4	<0.001
Marital status—married (%)	79.2	80.5	61.3	<0.001
Obesity (%) ^e^	40.9	41.0	39.9	0.149
BMI (kg/m^2^) *	24.6 (3.0)	24.6 (3.0)	24.5 (3.3)	0.013
hsCRP (mg/L) ^#^	0.05 (0.03–0.10)	0.05 (0.03–0.10)	0.05 (0.03–0.10)	0.766
Total vitamin D (ng/mL) ^#^	16.5 (12.7–21.3)	16.7 (12.8–21.4)	14.9 (11.5–19.2)	<0.001
PSA (ng/mL) *	0.91 (0.46)	0.91 (0.46)	0.92 (0.47)	0.592

Data are expressed as * mean (standard deviation), ^#^ median (interquartile range), or percentage. ^a^ ≥10 g/day. ^b^ ≥3 times/week. ^c^ ≥ College graduate. ^d^ Total monthly household income ≥$6000/month. ^e^ BMI ≥ 25 kg/m^2^. ^§^ *p*-value by chi-square test or *t*-test. BMI, body mass index; hsCRP, high-sensitivity C-reactive protein; PSA, prostate-specific antigen.

**Table 2:** Risk of elevated PSA according to shift work status.

Shift Work Status	Person-Years (PY)	Incident Cases	Multivariable-Adjusted HR (95% CI) ^a^				HR (95% CI) ^b^ in Model Using Time-Dependent Variables
			Age-Adjusted Model	Model 1 *	Model 2 **	Model 3 ***	
Daytime worker	252,841.7	970	1.00 (reference)	1.00 (reference)	1.00 (reference)	1.00 (reference)	1.00 (reference)
Shift worker	16,605.5	60	1.33 (1.02–1.72)	1.35 (1.03–1.78)	1.35 (1.03–1.77)	1.37 (1.04–1.80)	1.36 (1.04–1.79)

^a^ Estimated from Cox proportional hazard models. ^b^ Estimated from Cox proportional hazard models with smoking status, alcohol intake, regular exercise, sleep duration, BMI, hsCRP, and total vitamin D as time-dependent variables and baseline age, center, education level, total household income, marital status, shift work status, and weekly working hours as time-fixed variables. * Model 1 was adjusted for age, center, smoking status, alcohol intake, regular exercise, education level, total household income, marital status, and weekly working hours. ** Model 2: Model 1 plus adjustment for BMI and hsCRP. *** Model 3: Model 2 plus adjustment for sleep duration and total vitamin D. PSA, prostate-specific antigen; HR, hazard ratio; CI, confidence interval; BMI, body mass index; hsCRP, high-sensitivity C-reactive protein.

**Table 3:** Risk of elevated PSA according to shift schedule type.

Shift Schedule Type	Person-Years (PY)	Incident Cases	Multivariable-Adjusted HR (95% CI) ^a^				HR (95% CI) ^b^ in Model Using Time-Dependent Variables
			Age-Adjusted Model	Model 1 *	Model 2 **	Model 3 ***	
Daytime worker (*n* = 62,278)	252,841.7	970	1.00 (reference)	1.00 (reference)	1.00 (reference)	1.00 (reference)	1.00 (reference)
Fixed shift worker (*n* = 245)	978.5	4	1.11 (0.42–2.97)	1.07 (0.40–2.86)	1.07 (0.40–2.86)	1.09 (0.41–2.90)	1.08 (0.40–2.88)
Rotating shift worker (*n* = 3413)	11,857.7	42	1.39 (1.02–1.90)	1.45 (1.05–2.00)	1.44 (1.04–2.00)	1.47 (1.06–2.03)	1.46 (1.06–2.02)
Other shift worker (*n* = 1018)	3769.3	14	1.22 (0.72–2.06)	1.22 (0.72–2.08)	1.21 (0.71–2.06)	1.23 (0.72–2.09)	1.22 (0.72–2.07)

^a^ Estimated from Cox proportional hazard models. ^b^ Estimated from Cox proportional hazard models with smoking status, alcohol intake, regular exercise, sleep duration, BMI, hsCRP, and total vitamin D as time-dependent variables and baseline age, center, education level, total household income, marital status, shift schedule type, and weekly working hours as time-fixed variables. * Model 1 was adjusted for age, center, smoking status, alcohol intake, regular exercise, education level, total household income, marital status, and weekly working hours. ** Model 2: Model 1 plus adjustment for BMI and hsCRP. *** Model 3: Model 2 plus adjustment for sleep duration and total vitamin D. PSA, prostate-specific antigen; HR, hazard ratio; CI, confidence interval; BMI, body mass index; hsCRP, high-sensitivity C-reactive protein.

**Table 4:** Hazard ratios (95% CI) ^a^ for elevated PSA by shift schedule type subgrouped by age.

Subgroup	Shift Schedule Type				*p* for Interaction
	Daytime Worker	Fixed Shift Worker	Rotating Shift Worker	Other Shift Worker	
Age					0.552
<40 years (*n* = 43,638)	1.00 (reference)	1.01 (0.25–4.06)	1.33 (0.88–2.02)	0.94 (0.44–1.99)	
≥40 years (*n* = 23,179)	1.00 (reference)	1.17 (0.29–4.71)	1.74 (1.02–2.97)	1.74 (0.82–3.69)	

^a^ Estimated from Cox proportional hazard models adjusted for age, center, smoking status, alcohol intake, regular exercise, education level, total household income, marital status, weekly working hours, BMI, hsCRP, sleep duration, and total vitamin D. PSA, prostate-specific antigen; CI, confidence interval; BMI, body mass index; hsCRP, high-sensitivity C-reactive protein.

## Data Availability

The data are not available to be shared publicly, because we do not have permission from the Institutional Review Board to distribute the data. However, data can be available from the Kangbuk Samsung Health Study upon reasonable request, whose authors may be contacted through the corresponding authors for this manuscript.
